# Mechanical Stress Changes the Complex Interplay Between HO-1, Inflammation and Fibrosis, During Excisional Wound Repair

**DOI:** 10.3389/fmed.2015.00086

**Published:** 2015-12-15

**Authors:** Niels A. J. Cremers, Maarten Suttorp, Marlous M. Gerritsen, Ronald J. Wong, Coby van Run-van Breda, Gooitzen M. van Dam, Katrien M. Brouwer, Anne Marie Kuijpers-Jagtman, Carine E. L. Carels, Ditte M. S. Lundvig, Frank A. D. T. G. Wagener

**Affiliations:** ^1^Department of Orthodontics and Craniofacial Biology, Radboud University Medical Center, Radboud Institute for Molecular Life Sciences, Nijmegen, Netherlands; ^2^Experimental Rheumatology, Radboud University Medical Center, Radboud Institute for Molecular Life Sciences, Nijmegen, Netherlands; ^3^Department of Pediatrics, Stanford University School of Medicine, Stanford, CA, USA; ^4^Department of Surgery, University Medical Center Groningen, Groningen, Netherlands; ^5^Department of Plastic, Reconstructive and Hand Surgery, VU University Medical Center, MOVE Research Institute Amsterdam, Amsterdam, Netherlands; ^6^Association of Dutch Burn Centers, Beverwijk, Netherlands; ^7^Department of Orthodontics and Craniofacial Biology, Cleft Palate Craniofacial Center, Radboud University Medical Center, Nijmegen, Netherlands

**Keywords:** cleft palate, burns, mechanical stress, wound healing, heme oxygenase-1, inflammation, fibrosis

## Abstract

Mechanical stress following surgery or injury can promote pathological wound healing and fibrosis, and lead to functional loss and esthetic problems. Splinted excisional wounds can be used as a model for inducing mechanical stress. The cytoprotective enzyme heme oxygenase-1 (HO-1) is thought to orchestrate the defense against inflammatory and oxidative insults that drive fibrosis. Here, we investigated the activation of the HO-1 system in a splinted and non-splinted full-thickness excisional wound model using HO-1-*luc* transgenic mice. Effects of splinting on wound closure, HO-1 promoter activity, and markers of inflammation and fibrosis were assessed. After seven days, splinted wounds were more than three times larger than non-splinted wounds, demonstrating a delay in wound closure. HO-1 promoter activity rapidly decreased following removal of the (epi)dermis, but was induced in both splinted and non-splinted wounds during skin repair. Splinting induced more HO-1 gene expression in 7-day wounds; however, HO-1 protein expression remained lower in the epidermis, likely due to lower numbers of keratinocytes in the re-epithelialization tissue. Higher numbers of F4/80-positive macrophages, αSMA-positive myofibroblasts, and increased levels of the inflammatory genes IL-1β, TNF-α, and COX-2 were present in 7-day splinted wounds. Surprisingly, mRNA expression of newly formed collagen (type III) was lower in 7-day wounds after splinting, whereas, VEGF and MMP-9 were increased. In summary, these data demonstrate that splinting delays cutaneous wound closure and HO-1 protein induction. The pro-inflammatory environment following splinting may facilitate higher myofibroblast numbers and increase the risk of fibrosis and scar formation. Therefore, inducing HO-1 activity against mechanical stress-induced inflammation and fibrosis may be an interesting strategy to prevent negative effects of surgery on growth and function in patients with orofacial clefts or in patients with burns.

## Introduction

**Figure 1:**
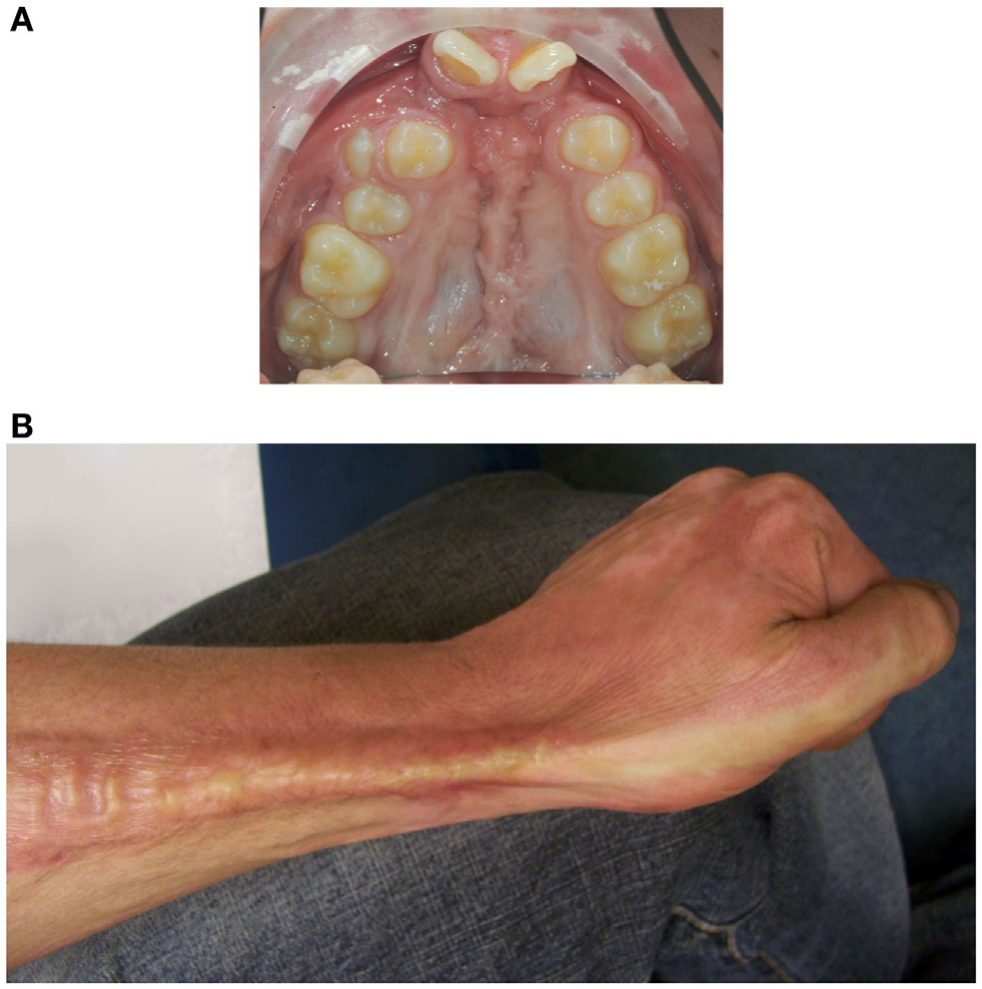
**Mechanical stress may promote excessive scar formation following injury**. **(A)** An intra-oral photo of the maxillary arch of a patient of our clinic with an operated complete bilateral cleft lip and palate is shown. Mechanical stress-induced scar formation impairs development of the upper jaw and the dentition. **(B)** Also, burns can result in excessive scar formation leading to cosmetic and functional problems as exemplified by scar formation near the wrist.

Cleft lip with or without cleft palate (CL/P) is a developmental craniofacial disorder that is characterized by an opening in the upper lip and/or palate and alveolar bone (1). Patients with CL/P need multiple surgeries that inevitably result in scar formation (Figure 1A) (2, 3). In particular, scars on the palate may disrupt normal midfacial growth and impair dento-alveolar development (4, 5). Also, patients with severe burns can exhibit excessive scar formation (Figure 1B) (6). Scarring can be exaggerated by mechanical tension, such as during growth of the child and during wound repair (7). Overall, pathological wound healing following mechanical stress can result in hypertrophic scars, and subsequently lead to functional, psychosocial, and esthetical problems for patients (8, 9).

Mechanical load, together with cytokine expression and the composition of the extracellular matrix (ECM), promotes the differentiation of fibroblasts into myofibroblasts (10–12). During wound repair, myofibroblasts play a key role in the deposition of ECM and in wound contraction, thereby reducing wound size and preventing invasion by pathogens (10, 12). When a wound closes, myofibroblasts normally disappear by apoptosis. However, during pathologic wound healing, extended presence of myofibroblasts may result in excessive wound contracture and ECM deposition, leading to excessive scar formation and functional problems (5, 13). Mechanical stress during wound repair can trigger a continued expression of the myofibroblast marker alpha smooth muscle actin (α-SMA) and a prolonged survival of myofibroblasts (5, 14–17). Prolonged inflammatory and oxidative stress may also increase myofibroblast survival (18). A better understanding of the effects of mechanical stress during the wound healing process is warranted to develop novel adjuvant therapies.

Rodents, in contrast to humans, possess a subcutaneous muscle layer (*m. panniculus carnosus*), which can cause wound contraction (19). In these animals, the use of splinting can induce static mechanical stress to healing wounds (5, 14, 20), interfere with muscle contraction, and thus better simulate human wound healing that is mainly dependent on granulation and re-epithelialization of tissues (19). The effects of static mechanical stress on the different phases of wound healing, caused by splinting, remains to be unraveled. In addition, the involvement of cytoprotective mechanisms needs further exploration. Activation of the cytoprotective heme oxygenase (HO) system has shown protective effects in both inflammatory and fibrotic models (6, 18, 21).

Heme oxygenase is an enzyme that catabolizes heme, yielding the gasotransmitter carbon monoxide (CO), free iron, which is scavenged by co-induced ferritin, and biliverdin that is rapidly converted into the antioxidant bilirubin by biliverdin reductase (22). The HO system possesses antioxidative, anti-inflammatory, anti-fibrotic, and anti-apoptotic properties (18, 23), and can influence cell proliferation, differentiation, and migration. When these processes are disrupted, wound repair may be hampered (24). There are two isoforms of HO, the inducible HO-1 and the constitutively expressed HO-2. It has been shown that HO-1 is rapidly induced in wounded tissues (25, 26). Decreased HO-1 or HO-2 expression and enzyme activity in mice results in slower cutaneous wound closure; whereas, induction of HO-1 expression or administration of the HO effector molecule bilirubin attenuates the inflammatory response and accelerates wound healing in HO-1-deficient mice (27–29).

In this study, we investigated the expression of HO-1 during wound repair in both non-splinted and splinted wounds using transgenic HO-1-*luc* mice. Because mechanical stress has been shown to induce HO-1 expression in different experimental settings in a time- and force-dependent manner (30–32), we therefore postulated that mechanical stress by splinting would induce HO-1 expression during wound healing. In addition, we investigated the effects of mechanical stress on markers of inflammation, ECM remodeling, and fibrosis.

## Materials and Methods

### Animals

The Committee for Animal Experiments of Radboud University Nijmegen approved all procedures involving mice (RU-DEC 2010-248). Twelve mice (strain: HO-1-*luc* FVB/N-Tg background), 4–5 months of age and weighing 30 ± 5 g, were provided with food and water *ad libitum*. Mice were maintained on a 12-h light/dark cycle and specific pathogen-free housing conditions at the Central Animal Facility Nijmegen. More details on the housing conditions have been previously described (33). Mice were originally derived from Xenogen Corporation (Alameda, CA, USA) and generated as previously described (34).

The mice were euthanized with a standard CO_2_/O_2_ protocol 7 days after wounding, after which control skin, and wounded skin were isolated. Half of the tissue was fixed for 24h in 4% paraformaldehyde, and then embedded in paraffin following regular histosafe procedures and the other half was snap-frozen in liquid nitrogen and stored at −80°C until isolation of mRNA.

### Excisional Non-Splinted and Splinted Wound Model

Splinted (*n* = 6) and non-splinted (*n* = 6) full-thickness excisional wounds 4 mm in diameter were created on the dorsum of the mice after shaving as previously described (35). In brief, excisional wounds were created using a sterile disposable 4-mm skin biopsy punch (Kai Medical, Seki City, Japan) on the dorsum to either side of the midline, and halfway between the shoulders and pelvis. Circular silicone splints of 6-mm inner- and 12-mm outer-diameter made from silicone sheets (3M, Saint Paul, MN, USA) were glued to the skin around the wound. Mice receiving splinted excisional wounds were wrapped with semi-permeable dressing (Petflex; Andover, Salisbury, MA, USA) around their torso, to cover the wound. One mouse in the splinted group died due to pulmonary failure as a result of respiratory obstruction by the bandage during the recovery from anesthesia.

Photographs of the wounds were taken immediately after wounding and then 1h and 1, 3, 5 and 7 days thereafter with a reference placed perpendicular next to the wounds for wound size normalization. The area of the wounds was blindly measured in triplicates using ImageJ (NIH) v1.44p software.

### HO-1 Promoter Activity Measurements

Heme oxygenase-1 promoter activity was determined at baseline, immediately after wounding and 1h and 1, 3, and 7 days thereafter in the HO-1-*luc*-Tg mice by *in vivo* bioluminescence imaging using the IVIS Lumina system (Caliper Life Sciences, Hopkinton, MA, USA) as previously described (36). Images were quantified using Living Image 3.0 software (Caliper Life Sciences) by selecting regions of interest (ROI). The amount of emitted photons per second (total flux) per ROI was measured, and then calculated as fold change from baseline levels.

### Immunohistochemical Staining

Immunohistochemical staining for HO-1, macrophages (F4/80), and myofibroblasts (α-SMA) were performed on paraffin sections of the wounds as previously described (35). In short, paraffin-embedded tissues were cut into 5-μm sections, which were then de-paraffinized, quenched for endogenous peroxidase activity with 3% H_2_O_2_ in methanol for 20 min, and rehydrated. Sections were post-fixed with 4% formalin, and washed with PBS containing 0.075 μg/mL glycine (PBSG). Antigens were retrieved with citrate buffer (0.01 M, pH 6.0) at 70°C for 10 min, followed by incubation in 0.075 g/mL trypsin in PBS at 37°C for 7 min. Next, the sections were pre-incubated with 10% normal donkey serum (NDS) in PBS-G. First antibodies (HO-1 from Stressgen #SPA-895 1:600 dilution, α-SMA from Sigma-Aldrich #A2547 1:600 dilution, and F4/80 from AbD Serotec #MCA497R 1:200 dilution) were diluted in 2% NDS in PBSG and incubated overnight at 4°C. After washing with PBSG, sections were incubated for 60 min with a biotin-labeled secondary antibody against host species (1:5000 dilution). Next, the sections were washed with PBSG and treated with avidin–biotin–peroxidase complex (ABC) for 45 min in the dark. After extensive washing with PBSG, diaminobenzidine–peroxidase (DAB) staining was performed for 10 min. After rinsing with water, staining was intensified with Cu_2_SO_4_ in 0.9% NaCl and rinsed with water again. Finally, the nuclei were stained with hematoxylin for 10s and sections were rinsed for 10 min in water, dehydrated and embedded in distyrene plasticizer xylene (DPX).

Immunoreactivity was evaluated by blindly scoring the wounds. For the HO-1 staining, we scored both the epidermal and dermal region of the wounds separately, since there were two different positively stained populations. A single section per wound was semi-quantitatively scored, by two assessors independently of each other, as previously described according to the following scale: 0 (minimal), 1 (mild), 2 (moderate), and 3 (marked) (35).

### RNA Isolation and Quantitative-RT-PCR

Non-wounded control skin and wounds were pulverized in TRIzol (Invritrogen) using a micro-dismembrator (Sartorius BBI Systems GmbH, Melsungen, Germany) and RNA was further extracted as previously described (37). Quantitative-RealTime-PCR was performed. mRNA expression levels were calculated as minus delta delta Ct (−ΔΔCt) values, normalized to the reference gene GAPDH, and corrected for non-wounded control skin. Fold change were calculated by 2^^(−ΔΔCt)^. The sequences of the mouse-specific primers are shown in Table 1.

**Table 1:** **Mouse primers**.

Marker	Gene name	Forward primer (5′–3′)	Reverse primer (5′–3′)
Reference gene	GAPDH	GGCAAATTCAACGGCACA	GTTAGTGGGGTCTCGCTCCTG
Inflammation	IL-1β	TGCAGCTGGAGAGTGTGG	TCCACTTTGCTCTTGACTTCTATC
	TNF-α	CTCTTCTCATTCCTGCTTGTG	GGGAACTTCTCATCCCTTTG
	COX-2	CCAGCACTTCACCCATCAGTT	ACCCAGGTCCTCGCTTATGA
	MCP-1	ACTGAAGCCAGCTCTCTCTTCCTC	TTCCTTCTTGGGGTCAGCACAGAC
Cytoprotection	HO-1	CAACATTGAGCTGTTTGAGG	TGGTCTTTGTGTTCCTCTGTC
	HO-2	AAGGAAGGGACCAAGGAAG	AGTGGTGGCCAGCTTAAATAG
Angiogenesis	VEGF	GGAGATCCTTCGAGGAGCACTT	GGCGATTTAGCAGCAGATATAAGAA
Fibrosis	α-SMA (acta2)	CAGGCATGGATGGCATCAATCAC	ACTCTAGCTGTGAAGTCAGTGTCG
	MMP-9	TGCCCATTTCGACGACGAC	GTGCAGGCCGAATAGGAGC
	Collagen 3a1	ATCCCATTTGGAGAATGTTG	AAGCACAGGAGCAGGTGTAG
Keratinocytes	Krt-6	GACGACCTACGCAACACC	AGGTTGGCACACTGCTTC

### Statistics

Data were analyzed using GraphPad Prism 5.01 software (San Diego, CA, USA). No outliers were detected using the Grubbs’ test. Data was analyzed by a paired *t*-test for comparisons of two groups and a one- or two-way analysis of variance (ANOVA) for the comparison of multiple groups with a *post hoc* Bonferroni correction for multiple comparisons. The non-parametrical one-tailed Mann–Whitney *U*-test was used to compare the arbitrary scored immunohistological sections. Results were considered significant different when *p* < 0.05 (*p < 0.05, **p < 0.01, ***p < 0.001).

## Results

### Static Mechanical Stress Induced by Splinting Delays Excisional Wound Closure

**Figure 2:**
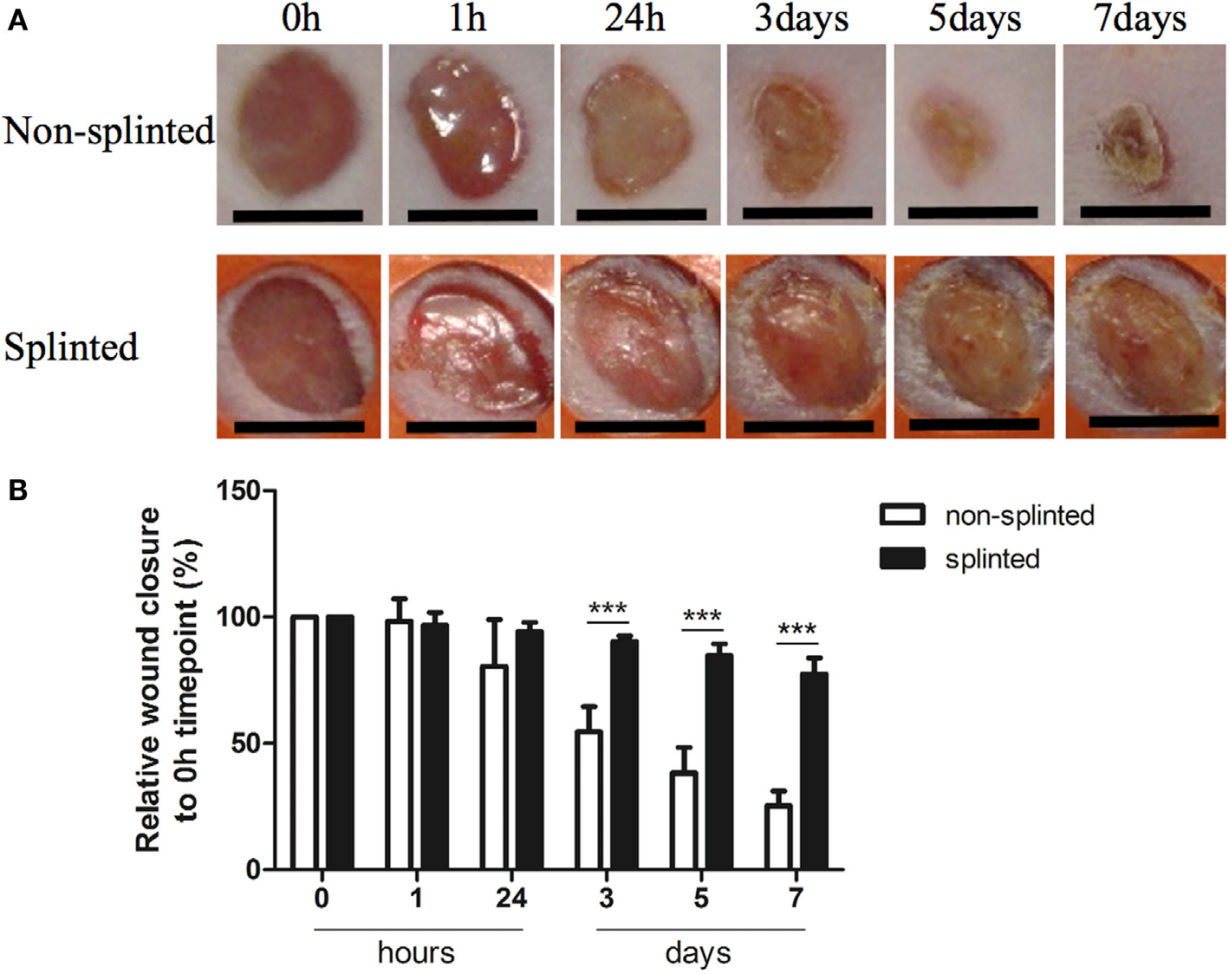
**Splinted and non-splinted excisional wound closure over time**. **(A)** Representative pictures of non-splinted and splinted wounds immediately following injury, and 1 h, and 1, 3, 5 and 7 days after wounding (black bars = 4 mm). **(B)** Quantification of closure of non-splinted (*n* = 6) and splinted (*n* = 5) wounds over time (0–7 days). Each mouse had two wounds. Time point 0 h was used as 100%. Data represent mean ± SD. *is significantly different between non-splinted and splinted wounds (***p < 0.001).

Because mechanical stress can lead to excessive scar formation (Figures 1A,B), we used both splinted and non-splinted excisional wound healing to assess the effects of static mechanical stress on wound closure. Wound sizes of non-splinted and splinted wounds were monitored over time and representative photos are shown in Figure 2A. After quantification of the wound area the wound closure time in relation to *t* = 0 h was displayed (Figure 2B). All wounds closed gradually, but there were differences in wound closure between splinted and non-splinted wounds. After 3 days, significant differences between the groups were found. Non-splinted wounds were already closed by 45%; whereas, splinted wounds had only closed by 10%. After 7 days, closure of non-splinted wounds was 75% of the wound area, while that for splinted wounds was only 23%. This corresponds to a 3.3 times faster wound closure rate when no mechanical stress was applied. This demonstrates that splinting effectively delays wound closure.

### The HO System is Affected by Mechanical Stress During Excisional Wound Healing

**Figure 3:**
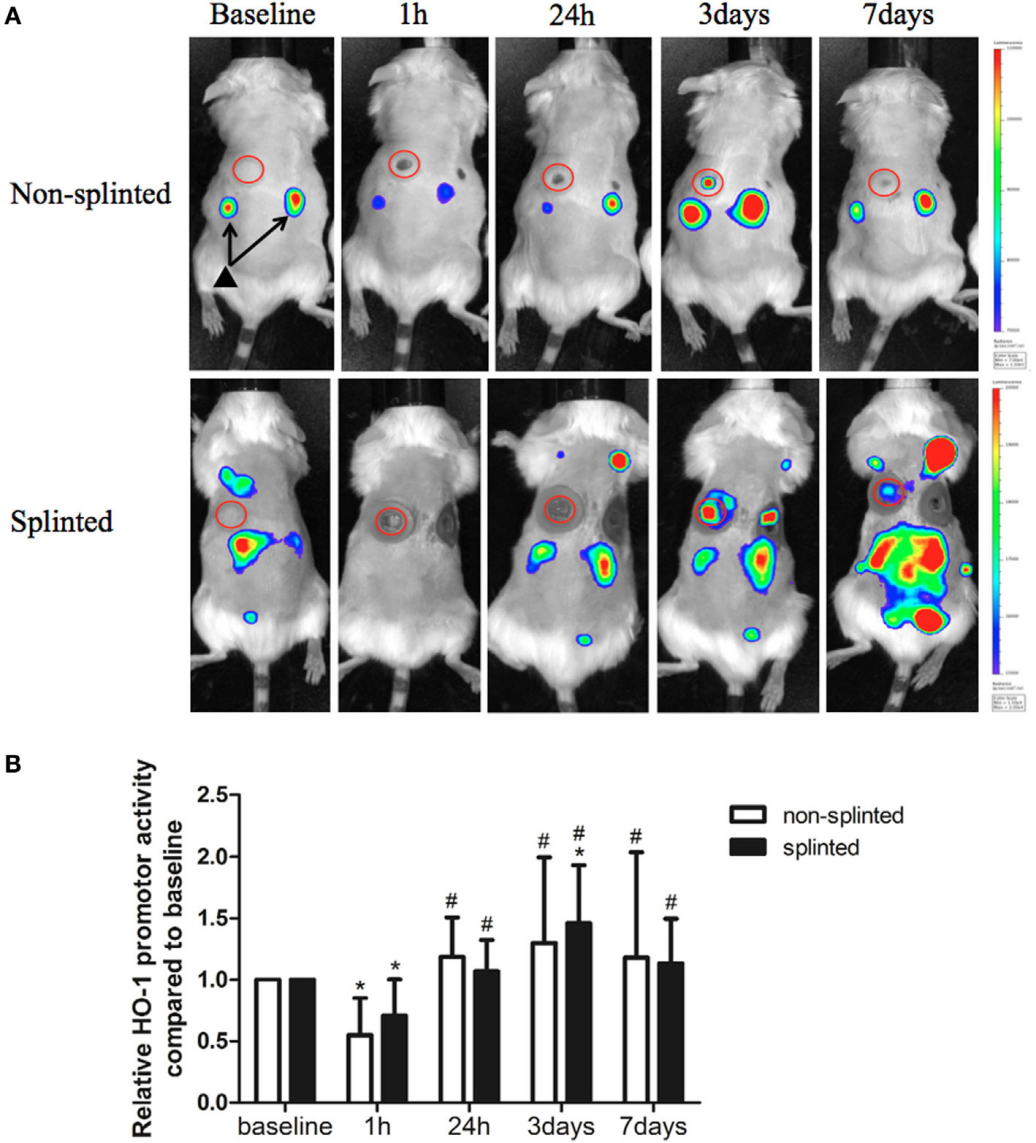
**HO-1 promoter activity in splinted and non-splinted wounds**. **(A)** Representative images of HO-1 promoter activity of non-splinted and splinted wounds over time using the IVIS system to measure *in vivo* bioluminescence in HO-1-*luc* mice. The rainbow-colored bar at the right side indicates the amount of luciferase signal represented in each group with a high signal in red and low signal in blue. The red circles surround the wounds in the mice and indicate the measured region of interest (ROI). Note: black triangle (▴) with black arrows shows that there is basal HO-1 promoter activity around the kidneys. **(B)** Quantification of HO-1 promoter activity during non-splinted (*n* = 6) and splinted (*n* = 5) wound healing. Each mouse had two wounds. Data represent mean ± SD. *is significantly different within the non-splinted or splinted group when compared to the baseline at the start of the experiment (*p < 0.05). #is significantly different within the non-splinted or splinted group when compared to 1 h after wounding (#*p* < 0.05).

Heme oxygenase-1 is thought to be a critical regulator and expressed in distinct cell types during the different phases of wound repair. In order to investigate the role of HO-1 following mechanical stress (splinting) during excisional wound healing, we used HO-1-*luc* mice to monitor HO-1 promoter activity (Figure 3A). HO-1 promoter activity was already present in the kidneys in non-injured HO-1-*luc* mice. In the wound area, HO-1 promoter activity was evident especially at days 3 and 7 after splinting.

Surprisingly, we found that following wounding, there was an initial significant decrease in HO-1 promoter activity after 1 h in both splinted as well as non-splinted wounds (Figure 3B). This decrease of HO-1 promoter activity returned to basal levels at day 1 and further increased at days 3–7, independent of splinting. Three days following wounding, the splinted wounds showed a significant increase of HO-1 promoter activity compared to basal levels.

**Figure 4:**
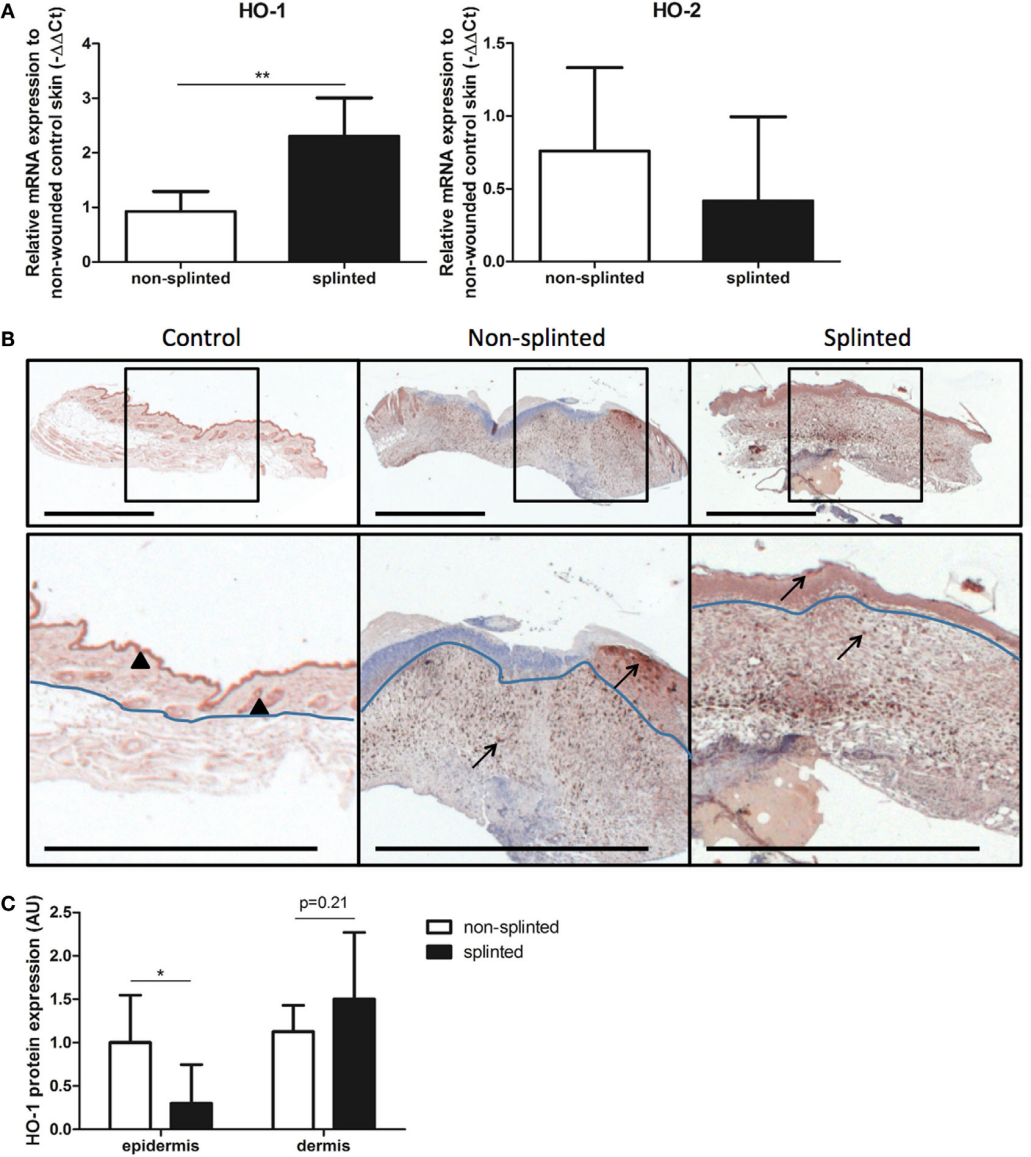
**HO expression in wounds**. **(A)** HO-1 and HO-2 mRNA expression levels in non-splinted and splinted wounds were determined in 7-day wounds and compared to control unwounded skin. Data represent mean ± SD. *is significantly different between non-splinted and splinted wounds (**p < 0.01). **(B)** HO-1 protein expression in control and non-splinted and splinted wounds after 7 days of healing. Region above the marked blue line is the epidermis and underneath the line is the dermal layer (bars = 1 mm). Note: black triangle (▴) in the non-wounded control skin shows HO-1-positive keratinocytes at the front of the epithelial layer, and HO-1 positive hair follicles. Black arrows (→) shows HO-1-positive cells in the epidermis and dermis of non-splinted and splinted wounds. **(C)** Quantification of scored HO-1 protein staining in epidermis and dermis of the wounds after 7 days in arbitrary units (AU). Data represent mean ± SD. *is significantly different between non-splinted and splinted wounds (*p < 0.05).

To further elucidate the role of splinting on HO-1 expression during wound repair, we measured both HO-1 mRNA and protein expression in day-7 wounds. Using RT-PCR, HO-1 mRNA expression was assessed in the wounds and corrected for expression in non-wounded control skin (Figure 4A). Here, we found significantly higher (2.6 times) HO-1 mRNA expression in splinted wounds compared to non-splinted wounds. As expected, the constitutively expressed HO-2 levels were not significantly different after induction of mechanical stress.

Immunohistochemical staining demonstrated that HO-1 levels were severely affected by wounding when compared to non-wounded skin. The number of HO-1-positive cells 7 days after wounding was increased in the dermal region of the wounds; also, more HO-1-positive cells were present in the epidermal region when compared to non-wounded skin.

Heme oxygenase-1 protein was particularly evident in the epithelial cells at the leading edge of the wound in the epidermis and in recruited inflammatory leukocytes in the dermis (Figure 4B). Since we observed two different populations of HO-1-positive cells depending on their region, the wounds were scored for the level of HO-1 protein expression in both the epidermal and dermal regions, which were compared between the different treatment groups (Figure 4C). Importantly, non-splinted wounds showed significantly higher HO-1 protein expression in the epidermal region compared to splinted wounds. In the dermal region, HO-1 was slightly higher after mechanical stress compared to the non-splinted wounds; however, this did not reach statistical significance (*p* = 0.21). Variation in HO-1 protein expression was found between animals, but was independent of the wound model.

Thus, wounding initially decreased HO-1 promoter activity in the wound, after which HO-1 levels were restored and further induced by recruitment of inflammatory cells and keratinocytes. Surprisingly, although splinted wounds delay HO-1 expression, as shown by an increased HO-1 promoter activity and HO-1 mRNA expression, HO-1 protein expression was, in contrast to in the dermis, lower in the epidermis when compared to non-splinted wounds.

### Interplay Between HO-1 and Inflammation in Non-Splinted and Splinted Wound Healing

**Figure 5:**
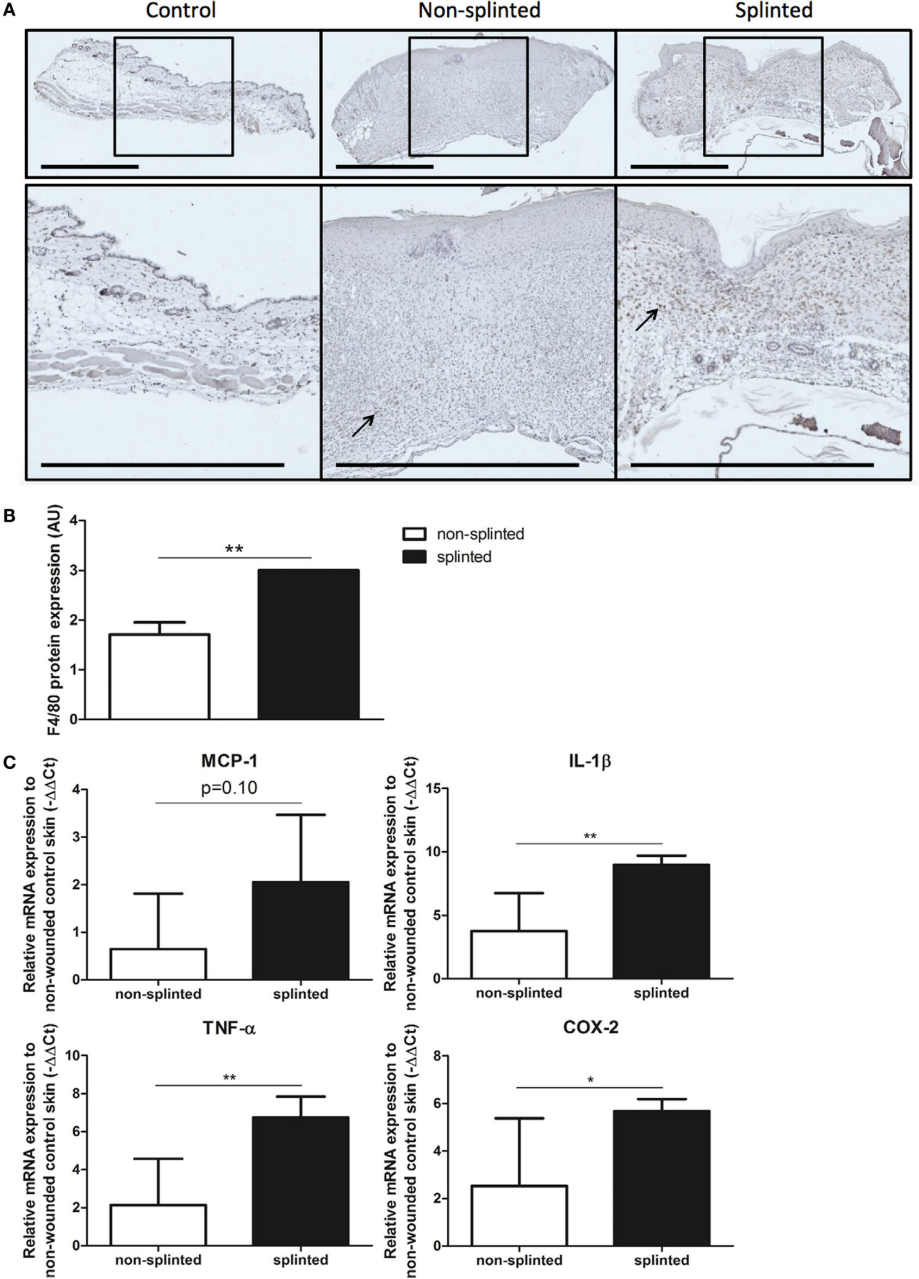
**Effects of splinting on markers of inflammation**. **(A)** Immunohistological staining of macrophages (F4/80) in control skin and non-splinted and splinted 7-day wounds (bars = 1 mm). Black arrow (→) shows F4/80-positive macrophages in non-splinted and splinted wounds. **(B)** Quantification of scored F4/80 protein staining of the wounds after 7 days in arbitrary units (AU). Data represent mean ± SD. *is significantly different between non-splinted and splinted wounds (**p < 0.01). **(C)** mRNA expression levels of inflammatory markers in 7-day non-splinted and splinted wounds normalized to levels in control unwounded skin (MCP-1, IL-1β, TNF-α, and COX-2). Data represent mean ± SD. *is significantly different between non-splinted and splinted wounds (*p < 0.05, **p < 0.01).

Since there was a clear delay in wound closure and HO-1 protein expression in splinted wounds, we investigated whether this delay correlated with altered levels of inflammatory gene expression. Because HO-1 has anti-inflammatory and antioxidative properties, we expected that the delayed HO-activity in the skin would result in increased levels of inflammation. Therefore, we quantitated the number of F4/80-positive macrophages using immunohistochemical staining in sections of non-splinted and splinted wounds (Figure 5A).

We found that there were indeed significantly more macrophages present in 7-day splinted wounds compared to non-splinted wounds (Figure 5B). Next, we investigated whether mRNA expression of monocyte chemotactic protein (MCP-1), the main chemokine to attract monocytes/macrophages, was altered by mechanical stress. We observed that MCP-1 was elevated 1.8-fold in splinted wounds compared to non-splinted wounds, however, this difference did not reach statistical significance (*p* = 0.10).

Finally, when we examined the gene expression of several other inflammatory markers, we found that mRNA expression levels of the pro-inflammatory cytokines IL-1β, TNF-α, and COX-2 in splinted wounds were significantly higher when compared to non-splinted wounds (36.9-, 24.4-, 8.8-fold, respectively) (Figure 5C).

In summary, we showed that delayed wound closure and reduced HO-1 protein expression following mechanical stress was clearly associated with elevated levels of macrophages and several other inflammatory genes.

### Effects of Mechanical Stress on Remodeling and Fibrotic Genes

Because increased levels of inflammation by mechanical stress during wound repair may also affect markers of fibrosis, we investigated the effects of splinting on markers of ECM remodeling and fibrosis.

**Figure 6:**
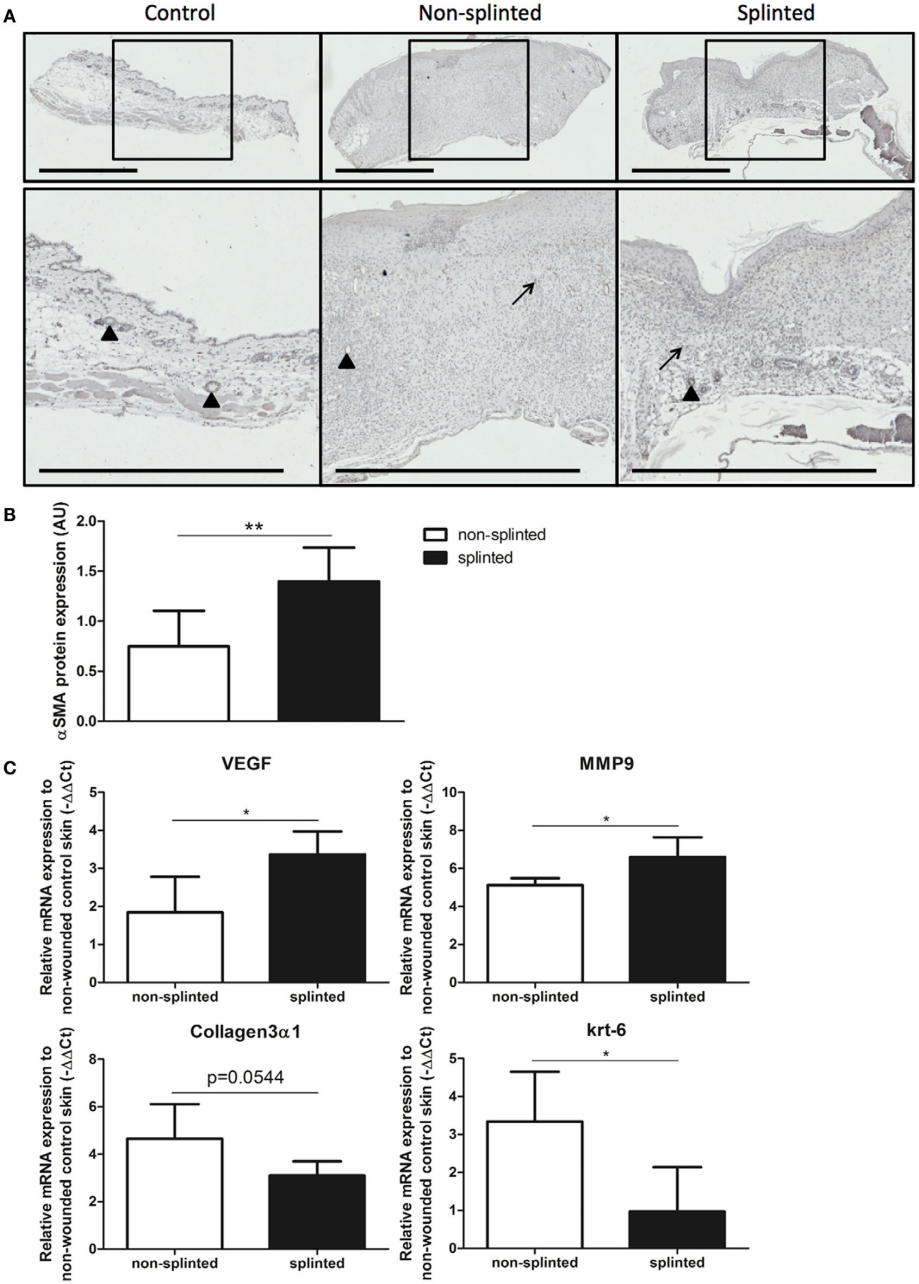
**Effects of splinting on markers of fibrosis**. **(A)** Immunohistological staining of α-SMA-positive myofibroblasts in control skin and non-splinted and splinted 7-day wounds (bars = 1 mm). Note: black triangle (▴) in the non-wounded control skin shows α-SMA-positive muscles in the vascular wall and the arrector pili muscles of the hair follicles. Black arrow (→) shows α-SMA-positive myofibroblasts. **(B)** Quantification of scored α-SMA protein staining of wounds after 7 days in arbitrary units (AU). Data represent mean ± SD. * is significantly different between non-splinted and splinted wounds (**p < 0.01). **(C)** mRNA expression levels of VEGF, α-SMA, MMP-9, collagen type 3, and krt-6 in 7-day non-splinted and splinted wounds normalized to levels in control unwounded skin. Data represent mean ± SD. *is significantly different between non-splinted and splinted wounds (*p < 0.05).

During wound healing, fibroblasts can transform into myofibroblasts. These myofibroblasts cause wound contraction and produce ECM. Normally, these myofibroblasts go into apoptosis after wound repair has finished. When these myofibroblasts fail to become apoptotic, ECM production continues, and pathologic wound healing with excessive scarring follows. The presence of myofibroblasts is dependent on the phase of wound healing. To investigate the role of splinting on myofibroblasts, we stained 7-day wound sections with the myofibroblast marker α-SMA. In non-wounded skin, α-SMA staining was already evident in the muscles of the vascular wall and the arrector pili muscles of the hair follicles (Figure 6A). Following injury, myofibroblasts were present both in non-splinted as well as splinted wounds. However, quantification of arbitrarily scored α-SMA-positive myofibroblasts showed significantly increased levels in splinted wounds when compared to non-splinted wounds (Figure 6B).

Next, we measured mRNA expression levels of genes involved in remodeling and fibrosis (Figure 6C). Vascular endothelial growth factor (VEGF) is an important regulator of angiogenesis, and was 2.9 times higher in splinted wounds compared to non-splinted wounds. Also matrix metalloproteinase (MMP)-9, an important enzyme that helps remodel the provisional ECM following wounding, was increased (four-fold).

Surprisingly, collagen type 3 gene expression, which is produced by (myo)fibroblasts, and forms the major collagen type expressed in granulation tissue, was almost three times lower in splinted wounds compared to non-splinted wounds (*p* = 0.0544).

Interestingly, we found significantly more (5.8-fold) gene expression of keratinocyte marker krt-6 in non-splinted wounds compared to splinted wounds.

Thus, there were more myofibroblasts and higher expression of VEGF and MMP-9 in day-7 wounds following mechanical stress.

## Discussion

We found that splinting effectively delayed wound closure. Interestingly, HO-1 promoter activity initially decreased upon wounding, but was followed by induction of HO-1 mRNA and protein. However, HO-1 protein expression was delayed in the splinted wounds. Since HO activity attenuates inflammatory and oxidative stress and is thought to reduce fibrosis, we postulated that the splinting-mediated delay in HO-1 protein expression could result in a reduced defense against inflammatory and fibrotic processes. Indeed, more F4/80-positive macrophages, α-SMA-positive myofibroblasts, and pro-inflammatory cytokines were present in splinted wounds at day 7 when compared to non-splinted wounds. Normally, the wound healing cascade is characterized by three distinct phases: inflammation, proliferation, and remodeling (9, 38, 39). This suggests that mechanical stress causes delayed wound closure, a prolongation of the inflammatory phase, and an altered remodeling phase, and may promote fibrosis.

### Mechanical Stress and HO-1

We found that mechanical stress by splinting delays wound closure 3.3 times in comparison to non-splinted wounds after 7 days. This was likely largely mediated by inhibiting the contraction of the subcutaneous muscle layer in mice; however, it may also be caused by interfering with other processes involved in wound healing, such as inflammation, proliferation or remodeling.

Previously, it was shown that *in vitro* administration of mechanical stress with a Flex-cell strain unit induces HO-1 mRNA and protein expression in periodontal ligament cells in a time- and force-dependent manner (30). HO-1 mRNA levels are also upregulated *in vitro* after mechanical stress in human aortic endothelial cells (40) and human dental pulp cells (41). Aortic smooth muscle cells also respond to mechanical stress, and induce HO-1 expression in a time-dependent response to laminar shear stress (32). In an *in vivo* model with inflammatory, oxidative, and mechanical stress, HO-1 mRNA was induced after 6 and 12 h as a result of unilateral urethral obstruction (31). We therefore postulated that splinting would induce HO-1 as a protective response.

We have shown that within the first hour of wounding, HO-1 promoter activity at the place of the wound decreases. This may be related to the removal of the HO-1-positive skin layer. This suggests that there is basal HO-1 promoter activity present in normal skin, which we previously validated in the epithelium of the skin (6). More precisely, keratinocytes in the hyperproliferative epithelium express high levels of HO-1 as normal physiology (25, 26). After the initial decrease in HO-1 promoter activity in both groups, HO-1 promoter activity significantly increased within one day, which may be due to the attraction of HO-1-positive macrophages during the inflammatory phase, and the influx of HO-1-positive epithelial cells during the re-epithelialization of the wounds (22, 26, 42). This rapid HO-1 induction is in line with a previous study in mouse excisional wounds, showing increased HO-1 mRNA and protein at day 1 after wounding (24). Additionally, the constitutively expressed HO-2 was also not affected by wounding in this previous study, which was similar to our current results. We found that effects on HO-1 promoter activity were independent of the wound model, since both splinted and non-splinted wounds showed similar activity. However, at day 3, there was only a significant increase in the splinted model compared to the baseline level. Increased HO-1 expression was also found for both models in 7-day wounds at the mRNA level when compared to non-wounded skin. This increase was significantly higher after mechanical stress.

On the protein level, we saw contradictory results with significantly more HO-1 protein staining in the epidermis of non-splinted wounds and a slight, non-statistically significant, increase of HO-1 protein in the dermis in splinted wounds after 7 days. Thus, although HO-1 induction was shown in diverse models of mechanical stress, we found only HO-1 promoter activation and HO-1 mRNA induction. HO-1 protein expression was, in fact, reduced by the static mechanical stress of the splint in the epidermal region.

When comparing protein levels in the epidermis, HO-1-positive cells were clustered in re-epithelialized tissue underneath the wound crust and were likely newly formed keratinocytes (25, 26). It is likely that the highly increased keratinocyte gene expression in non-splinted wounds account for the observed increase in HO-1 protein expression.

In the dermis, HO-1-positive cells in inflamed tissues were individually spread, and based on their location and morphology, appeared to be macrophages (25, 42). We and others demonstrated previously that HO-1-positive macrophages can be recruited during the wound repair process (6, 25, 26).

It is, however, striking that splinted wounds exhibited similar levels of HO-1-positive cells in the dermis when compared to non-splinted wounds, while there were higher levels of F4/80-positive macrophages in splinted wounds.

### Mechanical Stress by Splinting Enhances Inflammation; The Role of HO-1

Macrophages promote strong inflammatory responses through secretion of cytokines, such as IL-1β and TNF-α (43). It is thought that during the wound repair process, first pro-inflammatory M1 macrophages enter the wound site to clear cellular debris and destroy invading pathogens. Besides pro-inflammatory M1 macrophages, there are other macrophage subsets, including the anti-inflammatory M2 macrophages and the Mhem, Mox, and M4 macrophages (44–46). To enter the proliferation phase, resolution of inflammation is necessary. Under the right conditions, M1 macrophages may therefore skew into other anti-inflammatory macrophage subsets (45, 46). The presence of large numbers of macrophages in the splinted wounds and the increased levels of pro-inflammatory mediators IL-1β, TNF-α, and COX-2 suggests that in the splinted wounds there were mainly M1 macrophages present. Prolonged inflammation and high levels of oxidative stress may result in excessive deposition of ECM, leading to fibrosis and excessive/hypertrophic scarring (18, 47). Cytokines that mediate inflammation, such as IL-1β and TNF-α, are well known to be involved in mechanical stretch-induced inflammation. For example, *in vitro* administration of mechanical stress induces both IL-1β and COX-2 in fibroblast-like synoviocyte cells (48), epithelial cells (49), chondrocytes (50), and cardiac muscle cells (51) in a time- and force-dependent manner. This increased expression of inflammatory markers corresponded to our findings. Moreover, we found a trend in MCP-1 mRNA induction (*p* = 0.10) after mechanical stress, matching the enhanced protein staining for macrophages after mechanical stress.

Heme oxygenase-1-positive macrophages are thought to protect the wound environment against oxidative stress (25, 52). Following injury, the HO substrate heme is abundantly released at the edges of the wound site and can stimulate recruitment of leukocytes (6, 25, 53). In contrast, HO activity inhibits leukocyte recruitment via down-regulation of vascular adhesion molecules (6). Moreover, HO-1 activity inhibits production of various pro-inflammatory cytokines, including IL-1β and TNF-α (54, 55). In 7-day wounds, the inflammation levels were high in both non-splinted and splinted wounds, but however were much higher after mechanical stress in the splinted wounds.

The expression of HO-1 may facilitate resolution of inflammatory and oxidative stress at the wound site. When mechanical stress delays HO-1 protein expression, this may also delay resolution of inflammation, and lead to fibrogenesis.

### Mechanical Stress Results in More Fibrosis by a Prolonged Survival of Myofibroblasts; The Relation Between Inflammation, Remodeling, and HO-1

Mechanical stress, which is present in CL/P patients following palatal surgery and in patients with wounds in the vicinity of joints, increases the risk of excessive scar formation. In our splinting model, we demonstrated the increased presence of pro-inflammatory and pro-fibrotic cells and markers, suggesting that mechanical stress interferes with resolution of inflammation, which may ultimately lead to hampered wound repair and excessive scar formation. Application of mechanical loading to healing wounds in mice can cause hypertrophic scarring, through decreased apoptosis of myofibroblasts (56). It is tempting to speculate that the delayed HO-1 protein expression in the splinted model contributes to the delay in resolution of inflammation and allows the pro-inflammatory environment and the myofibroblast survival that we observed in the wound area at day 7.

When myofibroblasts fail to undergo apoptosis, this leads to continuation of contraction and production of ECM, and ultimately to fibrosis (5, 13, 17, 18). We found an increased presence of myofibroblasts after splinting, suggesting that either more myofibroblasts are formed upon mechanical stress, less myofibroblasts die, or splinting causes a delay in the myofibroblast formation when compared to non-splinted wounds. This corresponds to other reports, which showed that mechanical tension induces myofibroblast differentiation in wound granulation tissue in 6-day splinted wounds compared to non-splinted wounds (14, 20). These processes may be fine-tuned by HO-1 and its effector molecules as is shown *in vitro* by regulating the apoptosis of fibroblasts (18, 57).

Pathological cutaneous wound healing resulting in excessive scarring and fibrosis is the consequence of an imbalance between ECM synthesis by myofibroblasts and degradation and remodeling by MMPs (13). We found that remodeling marker MMP-9 was increased after mechanical stress, which was also observed by other groups (58–60). VEGF is an important promoter of angiogenesis and therefore microvessel density might be increased by mechanical tension. Chemokine and cytokine processing by MMPs affects the progression of inflammatory responses and leukocyte migration (61). For example, MMP-9 can inactivate/degrade CXCL1, CXCL4, CXCL9, and CXCL10, resulting in anti-inflammatory effects (62, 63). Increased MMP-9 may be indicative of inflammation and poor wound healing (64). The interplay between VEGF and HO-1 is complex and it was demonstrated that they can both inhibit or induce each other (65–68). Induction of HO-1 in a rat excisional wound model enhanced wound healing by increased cellular proliferation and collagen synthesis (28). HO-1 induction also reduced the inflammatory response by inhibition of pro-inflammatory molecules TNF-α, and ICAM-1 and an induction of the anti-inflammatory cytokine IL-10 (28).

In summary, mechanical stress leads to delayed wound closure, increased inflammation, and altered remodeling during the wound healing process. Since more myofibroblasts are present after splinting, mechanical stress may result in more scar formation. This was associated with a delayed anti-inflammatory and anti-fibrotic HO-1 protein expression in splinted wounds compared to non-splinted wounds. Since HO-1 attacks multiple targets that play an important role during fibrogenesis, pharmacologic induction of HO-1 may facilitate resolution of inflammation and attenuate fibrosis.

## Conclusion

We demonstrated that splinting significantly delays wound closure and potentiates the influx of pro-inflammatory leukocytes and myofibroblasts in day-7 wounds, which may promote fibrosis. The cytoprotective HO-1 gene is increased upon wounding, but HO-1 protein was lower in the epidermis after mechanical stress, probably as a result of the increased number of HO-1-positive keratinocytes in the re-epithelialization tissue of non-splinted wounds. Therefore, targeted pharmacologic induction of cytoprotective mechanisms, including HO-1, as preventive therapy against mechanical stress-induced inflammation and fibrosis must be considered.

## Author Contributions

NC, MS, MG, and CB performed the research. GD provided the original transgenic HO-1 *luc* mice for the study. NC, AK-J, CC, DL, and FW designed the study. NC, MS, MG, RW, KB, and FW analyzed the data. NC, RW, KB, AK-J, CC, DL, and FW wrote the manuscript. All authors approved the final version of the manuscript.

## Conflict of Interest Statement

The authors declare that the research was conducted in the absence of any commercial or financial relationships that could be construed as a potential conflict of interest.
